# Accelerating health system innovation: principles and practices from the Duke Institute for Health Innovation

**DOI:** 10.1016/j.patter.2023.100710

**Published:** 2023-03-22

**Authors:** Sahil Sandhu, Mark P. Sendak, William Ratliff, William Knechtle, William J. Fulkerson, Suresh Balu

**Affiliations:** 1 Duke Institute for Health Innovation, Durham, NC, USA; 2 Harvard Medical School, Boston, MA, USA; 3 Duke University School of Medicine, Durham, NC, USA; 4 Duke University Health System, Durham, NC, USA

**Keywords:** health innovation, machine learning, digital health, informatics, data science, informational technology, academic medical centers, organization and delivery of care, population health

## Abstract

The Duke Institute for Health Innovation (DIHI) was launched in 2013. Frontline staff members submit proposals for innovation projects that align with strategic priorities set by organizational leadership. Funded projects receive operational and technical support from institute staff members and a transdisciplinary network of collaborators to develop and implement solutions as part of routine clinical care, ranging from machine learning algorithms to mobile applications. DIHI’s operations are shaped by four guiding principles: build to show value, build to integrate, build to scale, and build responsibly. Between 2013 and 2021, more than 600 project proposals have been submitted to DIHI. More than 85 innovation projects, both through the application process and other strategic partnerships, have been supported and implemented. DIHI’s funding has incubated 12 companies, engaged more than 300 faculty members, staff members, and students, and contributed to more than 50 peer-reviewed publications. DIHI’s practices can serve as a model for other health systems to systematically source, develop, implement, and scale innovations.

## The bigger picture

Improving health care quality, costs, and access requires innovations in care delivery. Unfortunately, health systems often lack the full range of capabilities to foster and catalyze innovation efforts across the organization. In this perspective, we describe the principles and practices that have enabled the Duke Institute for Health Innovation to accelerate the development and implementation of health care innovations as part of routine clinical care within Duke Health. Future directions include sourcing and translating health care innovations beyond our own institution through research, commercialization, and public-private partnerships; further aligning innovation projects with emerging value-based payment structures; and informing regulatory policy for novel health innovations, such as machine learning products.

## Teaser

Despite the need for innovations in health care delivery to improve quality and reduce costs of care, health care systems often lack the capabilities and infrastructure to catalyze innovation efforts across the system. This perspective outlines the principles and practices that have enabled the Duke Institute for Health Innovation to accelerate the development and implementation of health care innovations as part of routine clinical care within Duke Health.

## Introduction

In recent years, the U.S. health care system has faced persistent and unprecedented challenges. Despite agreement on the need for reform, we have seen limited progress toward the quadruple aim of enhanced patient experience, improved health of populations, reduced total cost of care, and improved clinician well-being.^1^ The shift toward value-based care, coupled with technology breakthroughs, has created an opportunity to transform patient care and advance equity.

Academic health centers (AHCs) are well positioned to bring together the necessary building blocks to accelerate innovation. Although AHCs have historically focused on traditional forms of drug- or device-related biomedical innovation, they can also productize innovations in care delivery.^2^ These innovations aim to improve systems of care. AHCs benefit from transdisciplinary expertise, large clinical volumes and associated datasets, and diverse patient populations. They can re-design care for their own patients and staff members, while advancing the quadruple aim beyond their own health systems by training the next generation of clinician-innovators and commercializing innovations through university technology transfer offices. Unfortunately, AHCs may lack the full range of capabilities needed to systematically source, develop, and implement new care delivery solutions. Barriers include fragmentation across departments, faculty promotion criteria that reward traditional forms of scholarship, and high costs from caring for populations with complex health and social needs.^3^

One concrete mechanism to foster innovation through health systems has emerged in the past 10 years: innovations centers. As part of its 2015 survey, the Commonwealth Fund identified 67 such centers,^4^ defined as “places that are working to discover, develop, test, and/or spread new models of care delivery—in hospitals, clinics, and patients’ homes.” Innovation centers vary in their aims and vision, approaches for sourcing potential innovations, the types of digital technologies they commonly use, and the staff members and partners they engage. For example, Cleveland Clinic Innovations is more externally focused on supporting commercialization of proposed products by health system teams, while the University of Pennsylvania Center for Health Care Innovation is more internally focused on re-designing service delivery within existing business lines.^5^ A recent 2022 study concluded that health care organizations are largely still in the early stages of understanding what innovation centers can achieve, without clear consensus on optimal forms, goals, and impact.^6^

At Duke University and Duke Health, we launched the Duke Institute for Health Innovation (DIHI) in 2013. Recognizing that previous innovation efforts were siloed across academic units and service lines, we sought to create a platform to bring together interdisciplinary faculty members, staff members, and trainees across the university and health system. Critical to our implementation have been four guiding principles: build to show value, build to integrate, build to scale, and build responsibly. Our principles were informed by concepts from the business literature on the innovation value chain,^7^ the learning health systems framework,^8^ science and technology studies,^9^ and our own internal work on ethical and regulatory implications of data-driven innovations.^10^^,^^11^ Despite the spread of health care delivery innovation centers nationwide, few have synthesized insights across a large, sustained portfolio of work (more than 85 innovation pilots over 9 years) to provide a roadmap of steps to replicate and principles to operationalize into practice.

## Approach

### Build to show value

Building to show value requires innovators to work on the right problems with the largest potential for impact. Rather than focusing on quality improvement efforts that optimize existing services, we aim to support transformative innovations that lay the groundwork for future directions of our health system. We support projects that target diverse health conditions across the continuum of adult and pediatric care. The budget for innovation when DIHI was in startup phase in 2013 was about $880,000 and steadily grew to about $3 million. We received an annual operating budget from Duke Health to support individual projects and maintain a staff. Our director reports directly to the executive vice president of the health system and has a dual role as associate dean for innovation and partnerships reporting directly to the dean of the medical school.

Our annual request for applications (RFA) aligns both frontline staff members and organizational leaders. We first work with health system executives to identify four or five annual strategic priorities areas (e.g., population health and analytics, building resilience and well-being, enhancing transitions of care). We then use these identified priorities to publicize an annual RFA, to which frontline staff members and trainees can respond. An example of our RFA can be found in Appendix 1. Our proposal template includes ten sections: the problem; technology/intervention/process description; relevant background and prior work; proposed project; innovation; milestones, metrics, impact, and hurdles; risks and mitigation plans; funding request and use of funds; other assistance required; and references.

In the past nine years, we have received more than 600 proposals. Each proposal is reviewed by our own institute personnel, clinical and operational leaders and relevant subject matter experts (e.g., electronic health record [EHR] engineers). As a part of our due diligence process, we conduct assessments on project teams, feasibility, resource needs, and value to patients. Twenty finalists are selected to present oral pitches. Finalists work with our institute team to refine their presentation elements and meet with the health system’s technology support staff members to discuss any information technology (IT) integration requirements. Health system executives and clinical leaders select ten projects for funding amounts between $25,000 and $75,000 over a 12-month period.

Our method to funding projects is consistent with other “problem-driven” innovation approaches, in which unmet problems are first identified and potential solutions are then co-designed with frontline staff members in response to the problem.^12^ This contrasts with “technology-driven” or “opportunity-driven” innovation approaches, which start with a new technology and then attempt to apply and integrate it into real-world clinical settings. We consciously choose this approach for two reasons. First, given that we fund multiple projects each year, having all projects address the same set of strategic priorities set by our health system’s leadership ensures collective movement toward a shared set of organizational goals. Note that these strategic priorities evolve yearly in response to the most salient and timely problems the organization faces. Second, given that most of our technology solutions require engagement from frontline clinicians, we have consistently found that designing solutions that solve problems perceived as important to end users is critically important for adoption.^13^ Although some argue that problem-based solutions may incentivize incremental fixes over transformational care re-design, in our experience we have still been able to leverage novel technological tools (e.g., machine learning, augmented reality) in care delivery innovations (e.g., creating new types of workforce roles, delivering health care in untraditional settings such as the patient’s home).

### Build to integrate

In partnership with our awardees, we aim to build new solutions to integrate within routine clinical care. From the outset we require every team to receive sign-off from an operational leader who controls resources within the organization (e.g., hospital president, division chief, population health executive). Alignment with business unit leaders helps ensure that barriers encountered during the pilot are removed and that sustaining investment can be made available if the pilot is successful. This “living lab” approach allows innovations to be tested in relevant settings within the health system, rather than siloed clinical research units or innovation units separated from day-to-day care delivery.

In addition to sign-off for the project itself, teams often require their department leadership to provide additional financial support to cover protected time away from clinical or administrative responsibilities. This is because DIHI rarely funds specific line items to cover faculty or staff effort. Of note, maintaining this policy related to funding faculty effort did not decrease the annual number of applications in response to our RFA. However, some clinical departments submit more applications than others, because of higher levels of support from departmental or business unit leaders.

To facilitate project integration, we embed our own institute staff members on funded project teams. Drawing from design thinking and lean startup principles, our innovation project managers partner with funded teams to conduct a rigorous scoping process, in which we further refine the problem, engage relevant stakeholders, and define metrics. This process entails observing and interviewing frontline clinicians, mapping clinical workflows and care delivery processes, and creating patient journey maps. Unlike some incubators and accelerators that only fund and peripherally advise awardees, our own innovation project managers, together with project leaders, are accountable to health system leadership for the development, integration, and evaluation of the selected projects. Our project managers become the single point of contact for the project and drive progress and impact. Given that most projects include a technology and change management component, we purposefully recruit “pi-shaped” staff members (i.e., team members with deep expertise in at least two domains, often including business and technology).

Once project teams understand pain points, we build minimal viable solutions, an innovation approach in which we develop the simplest version of a product for end users to provide feedback to drive future iterations. Crucial to our solution development process is creating interfaces for transdisciplinary innovation. We actively engage and convene our own institute’s employees (e.g., solution architects and data engineers), clinical experts (e.g., frontline clinicians, operational leadership, nursing leadership), informational technology staff members (e.g., electronic health record developers and engineers), and quantitative experts beyond the health system (e.g., university faculty and staff members from computer science, engineering, and statistical science). Leveraging existing capabilities and expertise within our broader organization enables our institute to run lean with a small team of 11 full-time staff (organizational chart in Figure 1).

**Figure:**
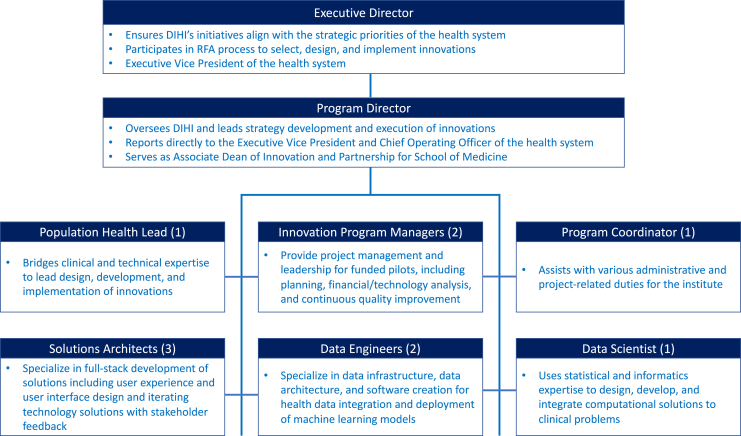
1Organizational structure

Although some innovation centers focus on a particular mode of innovation, such as mobile apps or telehealth, our approach recognizes that problems often require multi-component solutions that vary in scope and complexity: workflows, organizational structures, technologies, reimbursement, and internal policies and compliance regulations. For example, in our project to develop a sepsis early warning system for the emergency department, we developed and validated a machine learning algorithm that analyzed 42,000 inpatient encounters and 32 million data points. To ensure real-world uptake and avoid clinician alert fatigue, we displayed algorithm outputs on a user-friendly Sepsis Watch tablet application.^14^ With guidance from our project’s governance committee of physician, nursing, and administrative leadership, and frontline staff champions, we trained our hospital’s rapid response team (RRT) nurses to transition into a new Sepsis Watch nurse role to monitor the application and alert emergency department physicians of high-risk patients via phone.^15^ Our user interface designers met repeatedly with RRT nurses to iterate on functions, information, and visual components of the design. Preliminary results show that Centers for Medicare and Medicaid Services (CMS) SEP-1 bundle compliance increased from 31% between Q1 of 2017 through Q3 of 2018 to 64% between Q4 of 2018 and Q1 of 2020.

But not all projects succeed. Some projects fail in the implementation phase when there is a mismatch between benefits of a solution to the organization and benefits to the frontline end user. For example, research has shown that many patients with non-ST-segment-elevation myocardial infarction (NSTEMI) are at low risk for complications and do not require intensive care unit (ICU)-level care.^16^ Thus, leaders of our organization’s ICU had asked DIHI to create a decision support tool to identify lower risk NSTEMI patients in the emergency department to prevent overcrowding of the ICU.^17^ Unfortunately, this tool was overwhelmingly ignored by emergency department physicians. Discussions with the end user physicians revealed that they felt they did not need a “tool to tell them how to do their job.”^13^^,^^17^ The solution also required them to manually input data into the tool, outside of their normal workflow. In this example, emergency department physicians were required to contribute labor to adopt a tool that would ultimately benefit the ICU and the broader health care organization without addressing any of their own frontline problems.

### Build to scale

To accelerate our impact and build to scale, we focus on developing the foundational technical and workforce infrastructure to serve multiple innovations, within and beyond our health system. For example, in our project to identify patients at high risk for kidney failure in our Medicare accountable care organization, we implemented a predictive model that a multi-disciplinary team of clinicians could leverage during weekly “population rounding” meetings. When attempting to include the lab value for creatinine in our model, we found 14 distinct names related to creatinine in our EHR that needed to be grouped together.^18^ We realized that many of our own innovations and others in Duke’s broader learning health units required similar data cleaning and curation. Thus, we created a flexible data pipeline to reduce redundancy and waste across projects. This involved labeling, grouping, and harmonizing raw data elements, such as diagnosis codes, lab values, vital sign measurements, and medications. With readily available datasets for analysis, we can focus more of our efforts on solution development, implementation, and workflow design, thus accelerating the pace of innovations.

The process of scaling innovations within and beyond our institution has also surfaced multiple challenges. First, some technology solutions do not fit squarely within existing health system IT capabilities (e.g., mobile apps, augmented reality). DIHI innovations often expand IT capabilities, partly because we do not sit within an IT department, unlike innovation centers at other institutions, including Stanford and the University of California, San Francisco (UCSF). Nonetheless, being a separate organizational unit gives us more flexibility to become early adopters of new technologies. For example, we were the first group in our health system to use a new workflow management platform for data engineering. Over time, we helped build capabilities within our central IT department to maintain this technology.

Second, the heterogeneity of business models within our health system and flat reporting structure at senior leadership levels can hinder diffusion of an innovation. For example, an innovation that successfully reduces costs for our accountable care organization, if scaled across the institution, would reduce revenue for fee-for-service contracts. Finally, siloes across the organization hinder scaling of solutions. Innovations are often implemented in specific service lines, such as the emergency department or inpatient pediatrics, and departmental leaders who compete for resources may not efficiently adopt best practices from one another.

Beyond investments in technological infrastructure, we also invest in building a workforce of innovators across the health enterprise. We have found that our project awardees, who are often early career clinician-innovators, become sustained champions for innovation across the health system and beyond, helping create a culture of entrepreneurship and interdisciplinary collaboration. Our awardees have often brought in new faculty and staff members to adapt elements of their own projects to serve other patient populations or settings. Some of our innovators, after successfully implementing their projects, have been promoted to new operational leadership roles (e.g., associate chief medical officer for innovation and improvement, associate vice chair for inpatient operations), in which they can continue to scale their innovations and test new care delivery models.

Capacity building extends to trainees as well, as we support learners across various levels and disciplines. For example, our year-long, full-time clinical research and innovation scholarship program has prepared 30 medical students to lead the design, development, and implementation of health products and services.^19^ We have also engaged non-clinical learners through both curricular and co-curricular offerings. For example, we partnered with the Duke Social Science Research Institute to teach a semester-long undergraduate course on evaluating health innovations, in which 30 students developed measurement and evaluation plans for DIHI projects. We also created internship programs that have engaged more than 40 undergraduate and graduate students in data science and mobile application development projects between 2015 and 2022.

Having a dedicated and connected pool of innovators within our own institute and throughout our health system allowed us to quickly mobilize a response to the COVID-19 pandemic. Not bound to specific service lines or to frontline clinical responsibilities, our institute staff members pivoted to partner with faculty and staff members across the university to custom-build a Duke Symptom Monitoring app (SymMon) that all university students, faculty members, and staff members used to report COVID-19 symptoms before coming to campus each day.^20^ The app also facilitated pooled surveillance testing through a barcode scanner that linked specimens to persons and created labels for EHR orders. To serve communities outside our university, we partnered with nine community-based organizations, federal, state, and local public sector partners to launch the national Pandemic Response Network (PRN). Through our network, we provided web-based and telephonic support, in English and Spanish, to more than 10,000 users in 40 states to manage COVID-19 symptoms at home and facilitate connections to relevant medical and social services. Furthermore, through a partnership with IDEO.org, we identified key insights to remove barriers to reach populations that have been historically marginalized and launched programs to help public schools and small businesses to support vulnerable families.

### Build responsibly

Engaging with new domains of health care innovation such as artificial intelligence (AI) often presents unique ethical, legal, and moral challenges. Our institute team is committed to building responsibly by developing and disseminating best practices for innovation. Here, we outline our best practices to ensure responsible implementation of innovations, particularly machine learning solutions, across each phase of development, implementation, and evaluation.

#### Privacy and data protection

Developing machine learning models requires securely accessing patient data in a way that maintains privacy. For projects related to data from our own health system, all data curation and analysis is conducted through a highly protected virtual network space where approved users can work with identifiable protected health information. Through secure virtual machines, our team members can use statistical tools to analyze data. All requests to export data (e.g., tables, figures, codes, datasets) must go through an official honest broker, who ensures protected health information is not exported. This program was funded through Duke’s Clinical and Translational Science Award from the NIH and by the Duke University Health System.

For projects related to external model development (e.g., Jefferson Health’s validation of our Sepsis Watch algorithm), protected health information stays at the local institution. Rather than move data from one institution to another, we provide software containers that can be run locally at the partner site to populate databases, conduct analyses, or evaluate models. This approach allows us to move code among institutions without needing to move patient data.

Given the additional privacy considerations related to pooling data form multiple health care institutions, we are hoping to critically challenge the assumption in the field that centralizing data across sites results in higher performing models. In an experiment using models developed to predict post-operative complications, we demonstrate cases in which a hospital only using its own data leads to a more accurate model than when combining data from multiple hospitals.^21^

#### Systematic data quality assurance

Before model development, data quality assurance of real-world data from electronic health records is needed to ensure machine learning models have face validity. To guide efforts, we developed and validated a machine learning data quality assurance framework applied to data curated for 247,536 patients across five machine learning efforts.^22^ Our framework has three phases: data element pre-processing (e.g., grouping data elements that measure the same object and transforming various units of measurement into a single reference unit per data element), data quality checks (e.g., assessing every data element for completeness, conformance, and plausibility), and data quality adjudication (e.g., clinicians confirming whether data elements are fit for use and if alternative data sources or additional transformations are needed). As a result of this process, we removed or transformed an average of 23.4 data elements for each project. Details of our framework and generalizable practices can be found elsewhere.^22^

#### Rigorous model development and validation

We developed a repeatable, rigorous stage-gate process for developing and validating our machine learning models before integration into routine clinical care. Before models are developed, outcome measures are developed with clinical experts, and performance and improvement targets are set relative to the baseline of standard practice.^23^ Clinical leaders provide insight on the trade-off between performance gains and clinical interpretability, depending on the clinical problem. Algorithms are first trained on retrospective patient cohorts. We typically divide our cohorts into subsets for training, testing, and internal validation. Models are evaluated for their predictive performance on the basis of their statistical performance measures (e.g., sensitivity, false-negative rate, positive predictive value), workload measures (e.g., number of alerts per shift, time to evaluate per alert), and economic measures (e.g., cost per false positive, cost per false negative). When possible, we also conduct temporal validations using a more recent cohort of patients from a different time to assess robustness over time and separate cohorts from our community-based hospitals to assess external geographic generalizability beyond our quaternary academic hospital.^24^^,^^25^

If models pass our retrospective validations, we then integrate the algorithm into the EHR in a “silent mode” to prospectively evaluate the model with real-time data, in which the model is not exposed to clinicians or used in clinical care. At the end of our silent period, we compare our model outputs (e.g., risk scores for in-hospital mortality at the time of admission) to outcomes from individual patient encounters. Simultaneously, we finalize clinical workflows and user interfaces that present model outputs to end users. Select clinical leaders serve as beta testers to view delayed results and validate thresholds from model outputs (e.g., high-risk thresholds for post-operative complications).

#### Responsible integration and monitoring

Once our models are retrospectively and prospectively validated and clinical workflows and user interfaces are developed, we integrate our models into clinical workflows. Clinical end users must understand how, when, how not, and when not to incorporate model outputs into clinical decisions. For our machine learning projects, we create “Model Facts” sheets to communicate important model information.^26^ Similar to product information for food and drugs, our model label specifications include model name, locale, and version, summary of the model, mechanism of risk score calculation, validation and performance, uses and directions, warnings, and other information.

In addition to promoting transparency about our models to front-end users to ensure informed adoption, models must be monitored and maintained. For our Sepsis Watch project, we developed processes to ensure continuous feedback loops from end users to inform iterative improvements. These included semi-structured interviews, groups discussions, and options to submit written feedback through our web application. More long term, we invited end users to help design the evaluation to test the effectiveness of Sepsis Watch tool in order to build trust from frontline clinicians in future decisions to sustain or not sustain the project. We also created a governance committee of administrative, physicians, and nursing leadership to monitor effectiveness of the program.^14^ To further maintain accountability, we also assembled an external data monitoring board including expert clinical researchers and to oversee the safety and effectiveness of Sepsis Watch.^11^

#### Health equity considerations

We have been intentional in considering how our innovations may inadvertently exacerbate existing inequalities and have developed proactive strategies to mitigate unintentional harm. First, in recent years, we have aimed to prioritize equity-focused projects more explicitly in our RFA process. In 2021 and 2022, “advancing health equity” was one of our six thematic areas to which innovators could submit proposals. For example, we recently funded a project to develop a machine learning natural language processing model to better identify patients with peripheral artery disease, specifically with the aim to increase the proportion of non-White and socioeconomically disadvantaged peripheral artery disease patients treated by a specialist. Similarly, we funded a project to identify inequities in each stage of organ transplantation and creating community and internal pathways to reduce barriers to access.

Second, we have been careful to consider how bias in our data and machine learning algorithms may negatively affect model performance for historically marginalized groups. We frequently conduct subgroups analyses among subpopulations to establish baseline disparities within disease areas that we can monitor over time. For example, in our project to establish a prediction model to identify HIV risk, we purposefully created a separate model using a female-only cohort given that previous models failed to predict incident HIV cases among women.^27^^,^^28^

Third, we have aimed to scale our solutions beyond the ivory towers of our resource-rich institutions. For example, for our HIV risk prediction project, we are currently supporting our faculty innovators who received an NIH R01 grant to validate this model using clinical data from two southeastern Louisiana health systems, in areas with greater HIV burden and lower pre-exposure prophylaxis uptake. Our team is partnering with local community-based organizations to help reach community members most at risk for community infection.

#### Sharing best practices

Given that the implementation and regulation of health care machine learning products are in its earliest stages, we have been keen to share our learnings through multi-institutional coalitions (e.g., Machine Learning in Health Care) and policy venues (e.g., the National Academy of Medicine, the U.S. Government Accountability Office).^29^ Building on these collaborative cross-institutional relationships, we recently launched Health AI Partnership, funded by the Gordon and Betty Moore Foundation, to develop and disseminate best practices for health AI software procurement, integration, and maintenance.^21^

## Outcomes

Since our inception, we have funded and implemented more than 85 innovation projects, incubated 12 companies, engaged more than 300 faculty members, staff members, and students, and contributed to more than 50 peer-reviewed publications. Table 1 highlights examples of innovation projects, and their impact, funded and developed through our sourcing process and through other strategic partnerships. More detailed descriptions of supported projects, project leads, and their outcomes can be found elsewhere.^30^

**Table 1:** Examples of innovation projects supported to date

Project Name	Technology Innovation Domain	Setting	Description and Impact
Early Identification of Sepsis	machine learning/artificial intelligence	emergency department	Developed deep learning sepsis detection and management platform model (Sepsis Watch). Implemented clinical workflows for nurses to monitor Sepsis Watch dashboard across three Duke hospitals and alert attending physicians of high-risk patients. Improved average CMS SEP-1 bundle compliance by 110% at hospital A, 45% at hospital B, and 133% at hospital C two years before and after implementation. Technology licensed by external vendor for commercialization and scaling.
Early Identification of Cardiac Decompensation and Cardiogenic Shock	machine learning/artificial intelligence	cardiac floors	Analyzed, validated, and modeled six clinical cardiac decompensation phenotypes. Launched a real-time dashboard to display patients meeting or at risk for meeting those phenotypes. Piloting a multi-disciplinary cardiogenic shock team to respond to patients at high risk. Technology licensed by external vendor for commercialization and scaling.
Early Recognition of Pediatric Patient Deterioration	machine learning/artificial intelligence	general inpatient floors	Developed machine learning model that exhibited improved performance in predicting hourly risk for clinical deterioration in pediatric inpatients within 24–48 h compared with our current institutional standard of care. Designed a dashboard solution to display deterioration risk scores and clinical workflows to mobilize rapid response team.
Advance Care Planning Notifications	machine learning/artificial intelligence	general inpatient floors	Developed a machine learning model to identify hospitalized patients at high risk for mortality. Created workflow to initiate advance care planning with attending physician and multi-disciplinary care review with pharmacy and case management. Currently evaluating intervention through cluster-randomized controlled trial.
Surgical Complication Prediction	machine learning/artificial intelligence	inpatient surgical floors	Developed and validated model to predict complications and mortality after surgery. Created online calculator requiring input of nine data fields produce a risk assessment within the clinic environment. Project team formed company that raised more than $12.9 million in venture capital funding.
Emergency Department (ED) Patient Flow	machine learning/artificial intelligence	emergency department	Developed and validated a machine learning model to reliably predict the need for inpatient and intensive care unit admission for patients who present to the ED. Designed real-time visual dashboards with prediction scores for use by hospital patient placement teams and ED patient flow coordinator. Partnering with Singapore’s largest consortium of health care institutions to validate their admissions model on our data and our models on their ED data.
Chronic Kidney Disease (CKD) Improvement Project	predictive model	population health management	Deployed predictive models to produce reports of Medicare accountable care organization beneficiaries at high risk for rapid CKD progression and complications. Implemented “population rounding” approach in which multi-disciplinary care team review reports and select interventions to reduce risks. During the first nine months, 438 patients at high risk for ESRD were reviewed, and 84 patients were referred to a nephrologist. Scaled approach to serve non-Medicare populations and other clinical groups (e.g., endocrinology, palliative care).
Autism and Beyond	machine learning/artificial intelligence; mobile application/digital health	direct to patient	Designed iOS autism research app to test new video technology to analyze a child’s emotion and behavior. Partnered with researchers in China, South Africa, and Argentina to reach global audience. Over 1 year, 1,756 families with children aged 12–72 months participated in the study, completing 5,618 caregiver-reported surveys and uploading 4,441 videos recorded in the child’s natural settings. This work led to two R01-funded grants.
Mobile Care Plans for Children with Medical Complexity	electronic health record (EHR) tool; mobile application/digital health	complex care program	Developed solution for nurses to write mobile complex care plans that patient’s parent(s) reviews and proposes edits via online portal, with the final version available for all Duke clinicians in the EHR. In first year of rollout, 94% of eligible patients received 162 care plans, and 74% parents reviewed them online. Project informed publicly available software platform and mobile application that won the Health Resources and Services Administration (HRSA) Maternal and Child Health Bureau’s Grand Challenge competition.
Cancer Distress Coach	mobile application/digital health	direct to patient	Developed and tested Cancer Distress Coach, a mobile application to deliver cognitive-based therapies for end users to manage their symptoms of cancer-related post-traumatic stress. Pilot work resulted in an R01 grant from the National Cancer Institute.
Identifying Inequities in Access to Organ Transplantation	electronic health record (EHR) tool	population health management	Quantifying disparities at each stage of organ transplantation (referral, screening, evaluation, committee deliberation, and decision), and creating community and internal pathways to reduce barriers to access.
Remote monitoring for onco-primary care	electronic health record (EHR)tool	primary care	Developed, implemented, and evaluated an automated, asynchronous home blood pressure management workflow to improve blood pressure control and primary care physician’s engagement with patients on active chemotherapy. Pilot work resulted in an R01 grant from the National Cancer Institute.
PSA Screening Algorithm	electronic health record (EHR) tool	primary care	Developed a novel, evidence-based PSA screening algorithm, integrated into the EHR as a clinical decision support tool. Observed increase in the percentage of men who met screening algorithm criteria, from 49.3% pre-implementation to 68.0% post-implementation. North Carolina State Cancer Commission adopted the algorithm as the state’s recommended policy for prostate cancer screening.
Hospital-to-Skilled Nursing Facility (SNF) Care Transitions	telehealth	transitional care	Implemented weekly telehealth video conferences to facilitate multi-disciplinary and multi-institutional review of patients discharged from Duke hospitals to partner SNFs. Observed an 11% reduction in unplanned, all-cause 30-day readmissions compared with patients discharged to the pilot SNFs during the same time frame of the prior year.
Acute Hospital Care at Home	digital health	emergency department; general inpatient floors	Created workflows to identify emergency department and inpatient patients at low risk for deterioration to receive inpatient level care in their home, via tele-medicine, remote monitoring, and home care providers. Received CMS waiver to pursue reimbursement from Medicare. First patient discharged in April 2021.
Primary Care Access for Residents and Fellows	telehealth	employee health	Developed primary care video visit program and concierge scheduling service to allow trainees to access care without having to leave the hospital. Data from annual wellness surveys before and after implementation showed decrease in perceived barriers to access primary care (58%–31%) and in perceived delays to access primary care (27%–21%).
Voices of Duke Health	podcast	direct to patient	Created physical listening booth (i.e., recording studio) for patients, trainees, and medical professionals to share their stories through facilitated conversations and promote culture of well-being and resiliency. Fifteen podcasts were recorded, with more than 7,000 listens. Project selected as a winner of ABIM Foundation’s inaugural Trust Practice Challenge.

Although outcome measures are defined at the individual project level and relate to at least one aspect of the quadruple aim, future evaluations are needed to study portfolio-level effects on our health system and catchment population. Measures for these portfolio-level effects could be informed by broader health system quality scores reported to national quality programs and accreditation organizations (e.g., CMS Hospital Care Compare, Joint Commission accreditation, Leapfrog’s Hospital and Surgery Center Ratings).^31^ The impact of DIHI’s efforts unfortunately cannot be accurately quantified by looking at broad health system quality measures, because DIHI projects last 12 months and serve as initial validations of concepts within narrow clinical settings. Substantial operational investment is required beyond a DIHI project to scale concepts to a degree that can affect broad health system quality measures. Nonetheless, over the past 10 years, Duke University Hospital has seen improvements or continued success in a number of national measures related to health outcomes, utilization, and costs. For example, from the 2011–2014 reporting period to the 2018–2021 reporting period, Duke University Hospital experienced reductions in death rates for heart attack patients (from 13.1% to 11.2%), stroke patients (from 16.1% to 11.4%), and coronary artery bypass graft surgery (CABG) patients (from 3% to 2.2%). Similarly, during the same reporting period, the hospital-wide rate of readmission after discharge from hospital decreased improved from 15.5% to 14.9%, including for patients with heart attacks, pneumonia, CABG surgeries, and hip/knee replacements. In the context of these improvements, Medicare spending per beneficiary per episode of care has remained at less than the national average at the same ratio between 2011 and 2021 (0.97 at Duke University Hospital compared with the national average of 0.99). Although these measures provide helpful context demonstrating improvements in care at Duke Health, we cannot attribute these improvements specifically to DIHI.

## Next steps

Realizing the full potential of DIHI will require us to fully embrace collaboration within our own organization and partners across public and private sectors. First, we will need to continue to translate and scale our local solutions to other health care organizations through research and commercialization. Given that our operating budget initially relied solely on internal funding to support local development of solutions, scaling up our work in recent years has depended on new partnerships to source resources outside our own organization. Multiple requests from other health systems to adapt our technologies for their own patient populations and from foundations to disseminate our best practices for health system innovation have motivated our future directions. Thus, in the past three years, we have grown our external funding by more than 10 times to advance best practices in innovation, data science, artificial intelligence, and diffusion of innovations. In 2023 we are projecting that the external support for innovation research will be more than $2 million. For example, we have already partnered with a clinical research consortium to validate our Sepsis Watch program at other health systems and have licensed our algorithm to a health analytics company to enable scale globally.

Second, rather than only sourcing innovation projects from within our own organization, we will bolster how we source innovations from industry partners and systematically evaluate them for our own community and patient population. We are adapting the well-established structures and processes for academic-industry partnerships in the pharmaceutical and medical device space to experiment (e.g., clinical trials, technology transfers) with new care delivery innovations related to artificial intelligence and digital health.

Third, within our own organization, we are considering how our innovations can more directly drive payment reform. As our health system adopts new population-based and episode-based value-based payment models, we are expanding our strategic collaborations with internal and external operational and financial partners. For example, more than a dozen innovations have been built in partnership with the Duke Population Health Management Office and during the COVID-19 pandemic, we worked directly with CMS to obtain a waiver for a hospital-at-home acute care program. Efforts to quantify our return on investment at the project and portfolio levels will be crucial to ensure our long-term sustainability and buy-in from health system leadership, industry partners, and external investors. Nonetheless, we must balance financial sustainability with our mission to catalyze transformative innovations in health and health care, because many projects worthy of investment to advance the quadruple aim are ahead of reimbursement policy.

## Limitations

This case study has several limitations. First, although this case study presents principles and practices that have enabled innovation at Duke Health, the content may not be implementable or generalizable to other settings. The infrastructure and capabilities have evolved over nearly a decade and requires strong leadership support and alignment across the organization. Testing approaches to innovation across sites will require resources and coordination that are beyond the scope of activities for any single innovation team. We direct readers interested in cross-site innovation to national models, such as the Center for Medicare and Medicaid Innovation (CMMI).^32^

Second, our case study does not present a single, unified framework for evaluating all 85 innovation projects in a comprehensive fashion. Throughout the case study, we highlight published examples of project outputs and in the outcomes section, we present multiple ways in which projects have been successful, such as improving care quality, advancing health equity, and optimizing use of hospital assets. Unfortunately, each type of success has a different impact on the organization, and at the present time there is no single set of metrics for innovations. We hope that case studies like ours can contribute to future formalization of innovation evaluation methods.

Last, although we direct readers to existing published literature on health innovation programs,^4^^,^^5^^,^^6^ this case study is not a comprehensive review of existing and emerging programs. Further research will be required to identify and assess practices across sites to determine program effectiveness. That type of review would likely require novel primary data collection directly engaging innovation teams across sites, because published literature from individual sites is inconsistent. We recommend survey methodology to provide quantitative data on the distribution of characteristics and practices across innovation centers, coupled with qualitative approaches (e.g., semi-structured interview with program leadership and staff members) to identify the barriers and facilitators to successfully developing and sustaining innovation centers. Future studies may also benefit from consensus approaches, such as the Delphi method, that bring together a panel of experts to come to a consensus on defining innovation centers, metrics of success for innovation centers, critical components and practices related to success, and priority areas for research.^33^ We hope that future research can build on this case study to evaluate approaches more systematically across sites.

## Lessons learned and opportunities for replication

To achieve the quadruple aim and succeed under new payment reforms, AHCs must invest in the resources, capabilities, and infrastructure necessary to innovate. Although our steps to develop and grow DIHI were tailored to meet the unique needs and resources of our institution, we believe that other AHCs could replicate core components to achieve similar results.

First, we recommend that operational and/or academic leadership invest in a designated institute or center, with dedicated funds and staff members to coordinate innovation efforts across the organization. Beyond financial resources, a reporting relationship to senior executives and visibility into health system operations can ensure alignment of innovation with strategic priorities. Similarly, our academic relationship with the medical school and broader university has been critical: we leverage their office of research administration and research contracts to bring in federal and foundation research funding; our physical co-location with the Duke Clinical Research Institute has facilitated close collaboration with experts in regulatory affairs and clinical trials; and we work closely with the university’s technology transfer office to protect inventions and negotiate licenses with external commercial partners interested in scaling our technologies. AHCs with fewer available resources should explore collaborative development opportunities with industry partners or other AHCs to build internal capacity.

Second, to maximize value created and drive culture change among frontline clinicians, we recommended that other AHCs replicate our model for crowdsourcing innovations. We have already started supporting community health centers interested in replicating our RFA process. We recommended that the RFA require a frontline clinician to part of the project team to ensure the project who can help shape the innovation to be most useful and feasible to be integrated clinical workflows.

Third, AHCs should hire and train staff members with expertise in design thinking, lean methodology, product development, and data analytics to support frontline clinicians in the implementation of their pilots.

Fourth, for machine learning and artificial intelligence projects, we are often able to hand off long-term maintenance of the project to the appropriate frontline clinical and operational teams. However, we recommend developing strategies to monitor, maintain, and update the algorithms themselves. Approaches include continuous monitoring of all model inputs and outputs, at least bi-annual assessment of model performance, and development of new algorithms for adjacent use cases and when model performance deteriorates.

Our practices and learnings can serve as a model for other health care organizations to develop the centralized infrastructure and a community of engaged innovators to systematically source, develop, implement, and scale innovations.

## Supplemental information

Document S1. Appendix 1

Document S2. Article plus supplemental information
