# Supplementary material for: Accelerating health system innovation: principles and practices from the Duke Institute for Health Innovation
Source: Patterns (N Y). 2023 Mar 22;4(4):100710. doi: 10.1016/j.patter.2023.100710 (PMC10140606; doi:10.1016/j.patter.2023.100710)
Supplement: Document S1. Appendix 1 [file mmc1.pdf]

Patterns, Volume *4*

Supplemental information

Accelerating health system innovation: principles and practices from the Duke Institute for Health Innovation

Sahil Sandhu, Mark P. Sendak, William Ratliff, William Knechtle, William J. Fulkerson Jr., and Suresh Balu

Duke Institute for Health Innovation

Catalyzing transformative innovations in health and healthcare

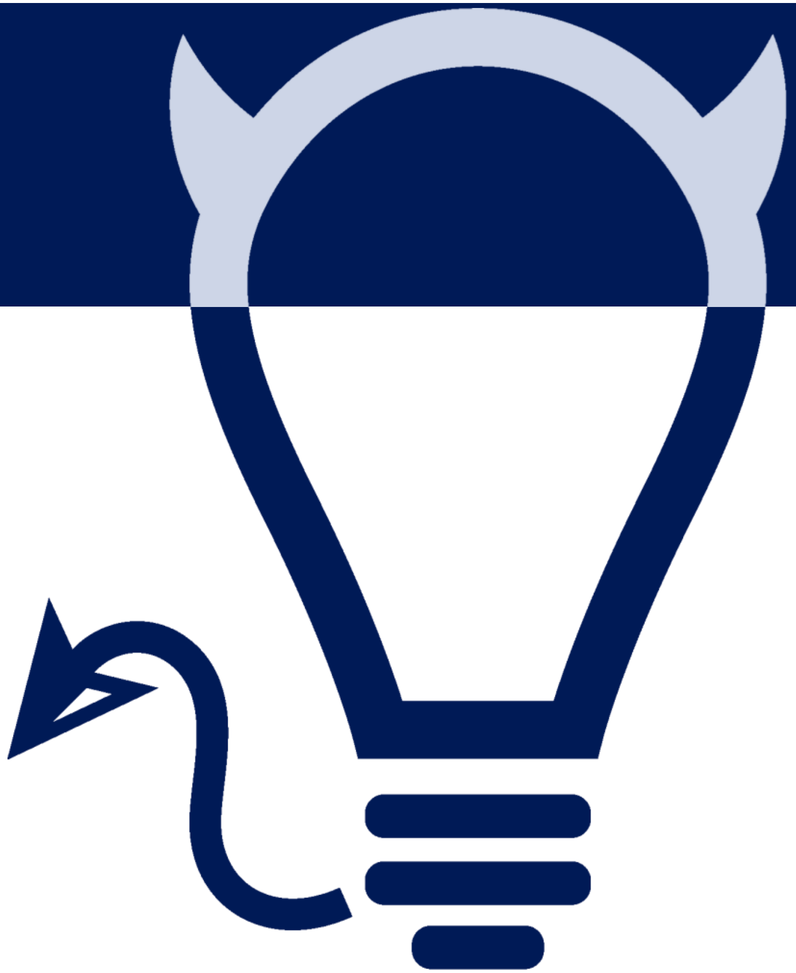

DIHI RFA 2022

Request for Applications (RFA) for Innovation Projects

Applications due 11:59pm on Friday, October 1, 2021

https://dihi.org/events/dihi-rfa/

Seeking Innovations in Health and Care Delivery

The Duke Institute for Health Innovation (DIHI) announces the next emerging ideas and innovation funding cycle. Applications are open to faculty, staff, trainees and students of Duke University and Duke University Health System and are due by **11:59PM Friday, October 1, 2021.**

Proposed innovation projects should address actual and important problems encountered by care providers, patients and their loved ones in our clinical enterprise and represent urgent health challenges nationally.

For the upcoming funding cycle, priority will be given to ideas aligned with these thematic areas:

- Advance health equity
- Enhance patient and community engagement and experience
- Accelerate population health strategies and solutions
- Improve value of care through novel strategies
- Grow digital care pathways and remote monitoring solutions
- Enhance care team experience and well-being; reduce workload

Seeking Innovations in Health and Care Delivery

Up to ten applications will be selected for support, with a total budget allotted for all selected projects not to exceed $450,000. Most proposals are expected to request funding in the range of $25,000 to $70,000 over a one-year period.

DIHI 2022 RFA TIMELINE	
Aug 2, 2021	DIHI RFA open for submission
**October 1, 2021 11:59PM**	**Application due date**
Feb 2022	Finalists – Presentation to leadership
Apr 2022	Funding start
Nov 2022	Midpoint reporting

Please forward this announcement to your collaborators and others who might be interested in applying. If you have any questions, please email DIHIrfa@duke.edu

Please visit **https://dihi.org/events/dihi-rfa** for more information on how to submit your application.

Proposal Template

Proposals should use the following section headings and adhere to a strict three (3) page limit, excluding references. Margins should be a minimum of 0.5" all around and font size no smaller than Arial 11 pt (aside from tables and figures). Please visit dihi.org/events/dihi-rfa for more information on how to submit your application. The proposal template is the following:

Problem: What problem(s) does the idea solve? Why is it a problem? Who is affected by and how large is the problem? How is the problem being dealt with today? How is this aligned with the DIHI RFA priorities and other Duke Health initiatives?

Technology/Intervention/ Process Description: In one or two sentences, describe the innovation. Please be concise, but also clear. Use simple terms; assume the reader is not an expert in the field.

Relevant Background and Prior Work: Describe your individual or team experience as it relates to understanding the current problem and developing/implementing your proposed innovation.

Proposed Project: What are you proposing to do? How does the innovation (technology, intervention, or process innovation) work to solve the target problem? Tell us a little bit about the clinic or part of the Health System where you would propose testing your idea? How would this be implemented in the proposed setting? Give us a sense for the relative magnitude of your solution. Does your solution completely eliminate the problem, or does it solve it partially?

Innovation: In one or two sentences, why is what you are proposing innovative?

Milestones, Metrics, Impact and Hurdles: What are the significant milestones and timeline during the grant period? (The term "grant period" refers to the period of time during which the awarded grant money will be spent, or one year.) Include target dates for each milestone and use measurable milestones only.
Identify the quantifiable success metrics for this project. What specific outcomes will you be assessing?

Risks and Mitigation Plans: What two to three specific hurdles or risks do you foresee that must be addressed for this project to succeed?

Funding Request and Use of Funds: For a one-year period, how much funding are you and your team seeking? What is the intended use of the requested funds? What business questions are you trying to answer and how will winning a grant help you find the answer(s) to these questions? Please be specific.

Other Assistance Required: In order for the proposed innovation to be successful, what other specific nonfinancial needs would you have?

References: No more than 20 (does not count against page limit).
